# Supplementary material for: Miniature computational spectrometer with a plasmonic nanoparticles-in-cavity microfilter array
Source: Nat Commun. 2024 May 7;15:3807. doi: 10.1038/s41467-024-47487-y (PMC11076628; doi:10.1038/s41467-024-47487-y)
Supplement: Supplementary file 1 — Supplementary Information [file 41467_2024_47487_MOESM1_ESM.pdf]

Supplementary Information for

Miniature computational spectrometer with a plasmonic nanoparticles-in-cavity microfilter array

Yangxi Zhang[1, †], Sheng Zhang[2, †], Hao Wu[1], Jinhui Wang[1], Guang Lin[2, 3,*], and A. Ping Zhang[1,*]

[1] Photonics Research Institute, Department of Electrical Engineering, The Hong Kong Polytechnic University, Kowloon, Hong Kong SAR, China.

[2] Department of Mathematics, Purdue University, West Lafayette, Indiana, USA.

[3] School of Mechanical Engineering, Purdue University, West Lafayette, Indiana, USA

[†] Y. Zhang and S. Zhang contributed equally to the work. [*] Corresponding author's email address: azhang@polyu.edu.hk, guanglin@purdue.edu.

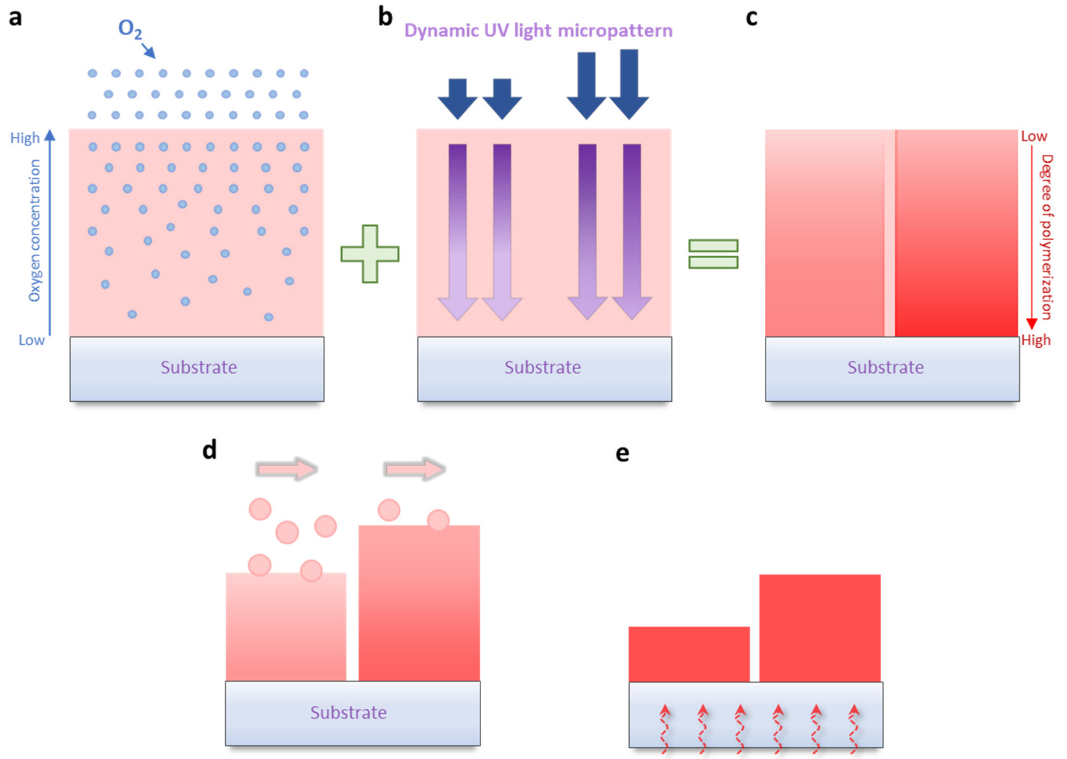

Supplementary Fig. 1 | Schematic diagrams and flowchart of digital grayscale photopolymerization technology for direct printing of length-varying polymer FP cavities. a, Formation of a gradient of oxygen inhibition degree by the diffusion of oxygen from the air into the film. **b,** Exponential attenuation of UV light due to absorption. **c,** Formation of a gradient of polymerization degree that is greater at the bottom. **d,** Creation of length-varying polymer FP cavities by the development of a less polymerized oligomer. **e,** Hard baking process.

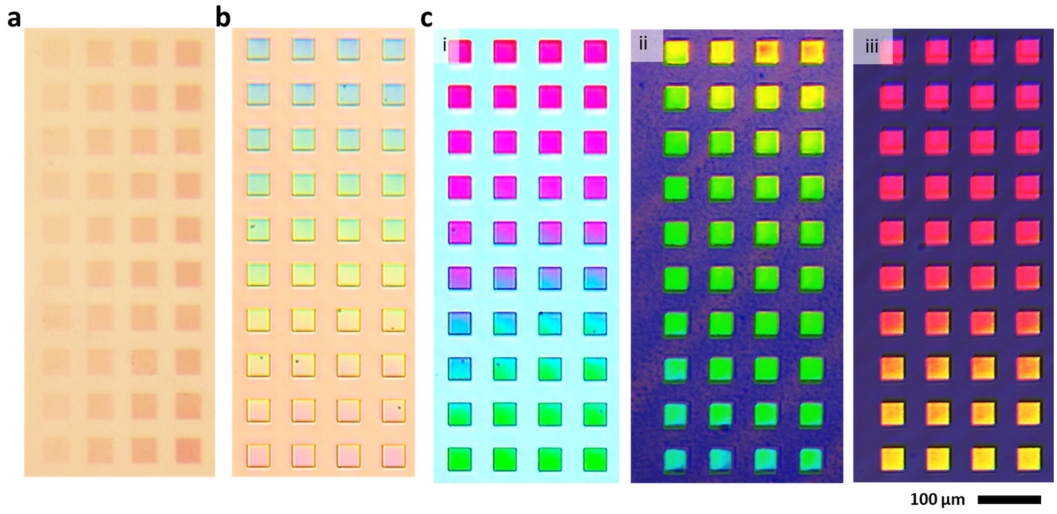

Supplementary Fig. 2 | Colour transmission optical microscopy images of the fabricated samples at different stages. a, Optical microscopy images after printing the micropatterns of the printed AgNPs on a TiO_2 layer (as well as the bottom Ag mirror and protective silica layers). **b,** Optical microscopy images after printing the polymer FP microcavities. **c,** Optical microscopy images of three groups of plasmonic nanoparticles-in-cavity microfilters fabricated from different sizes of AgNPs and FP cavities of different lengths. The scale bar is 100 µm.

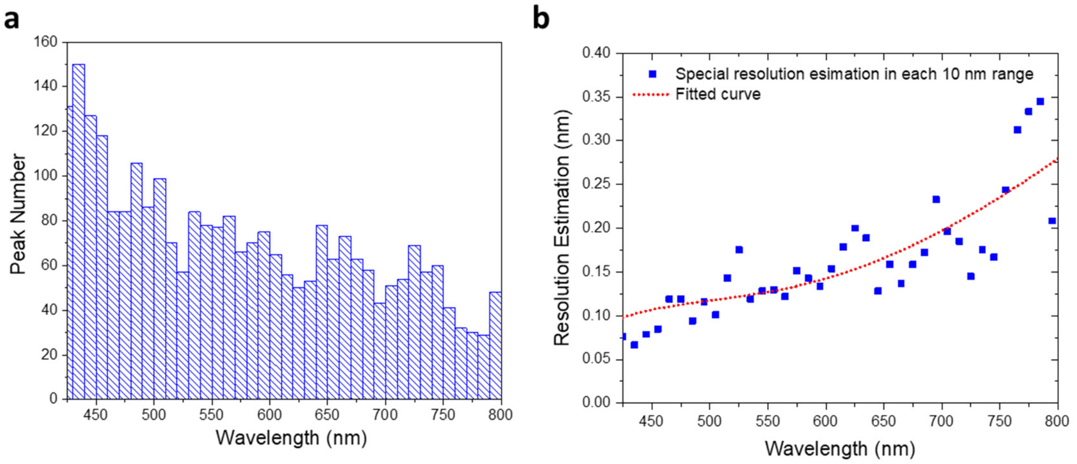

Supplementary Fig. 3 | a, Distribution of the transmission peaks of a fabricated plasmonic microfilter array. There are 2436 transmission peaks in the wavelength range from 425~800 nm. **b,** Estimation of the ideal spectral resolution using the transmission peak distribution.

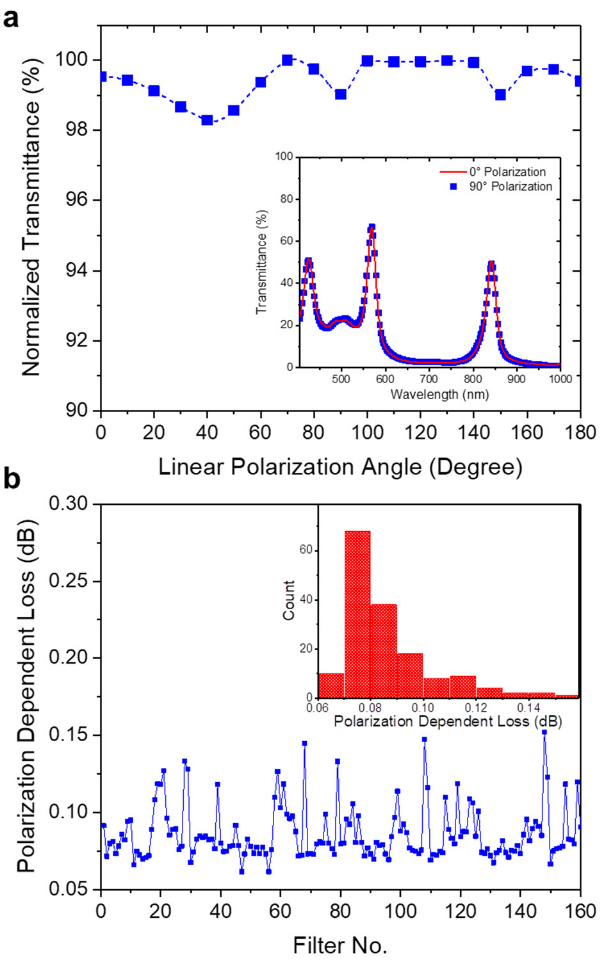

Supplementary Fig. 4 | Polarization-dependence measurement results for plasmonic microfilters. **a.** Normalized transmittance of a typical filter for 0° to 180° linearly polarized light. The inset shows the transmission spectra of a typical filter for 0° and 90° linearly polarized light. **b.** Measured polarization dependence loss of 160 filters. The inset is the statistical distribution of the polarization-dependent loss.

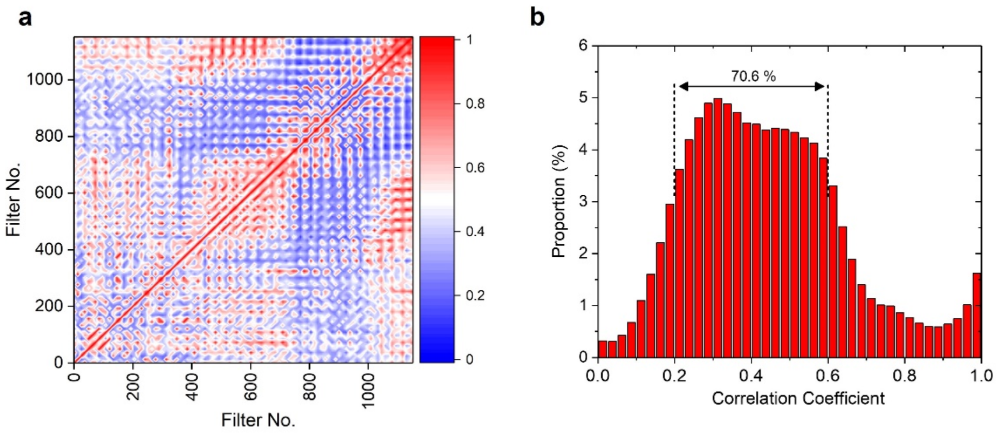

Supplementary Fig. 5 | a. Cross-correlation coefficients of the transmission spectra of a fabricated plasmonic microfilter array. **b**, Statistical distribution of the cross-correlation coefficients.

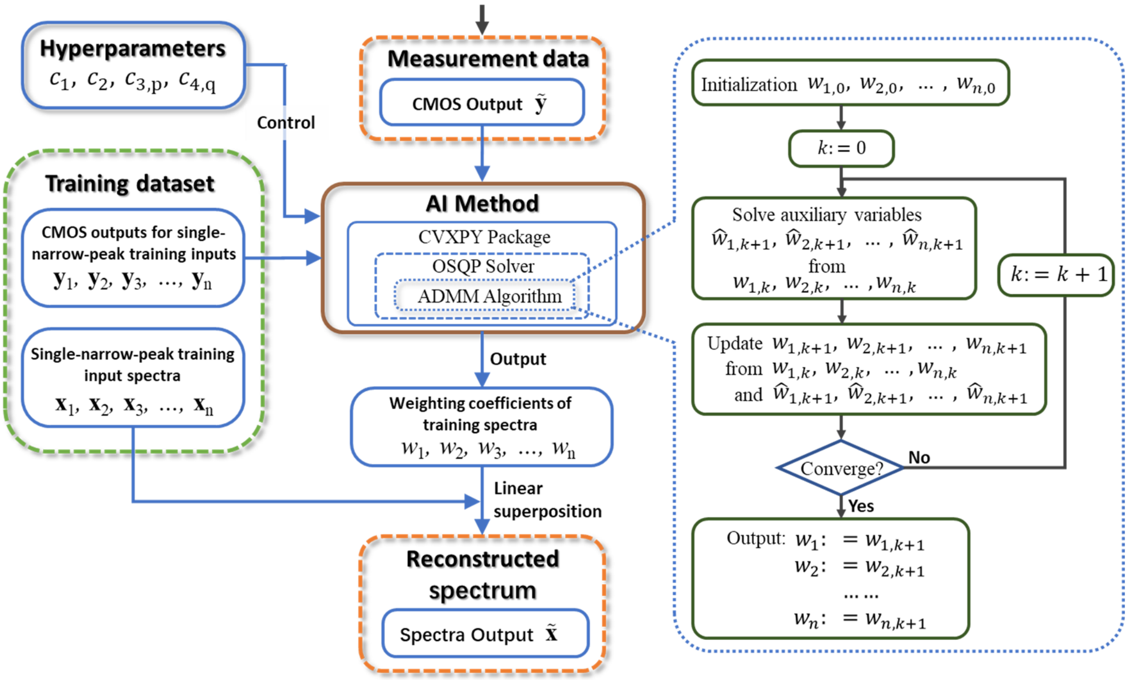

Supplementary Fig. 6 | Diagram of the machine learning-based AI method for spectrum reconstruction.

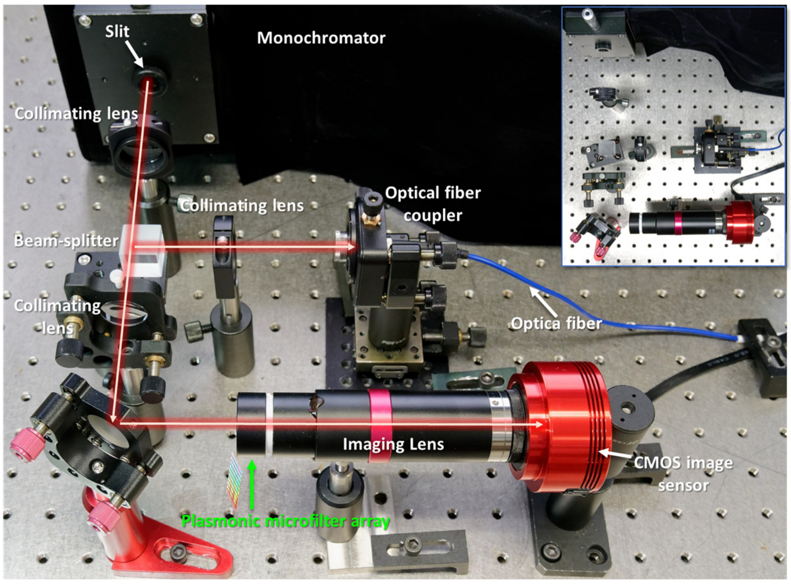

Supplementary Fig. 7 | Experimental setup for training and testing the plasmonic microfilter array-based computation spectrometer.

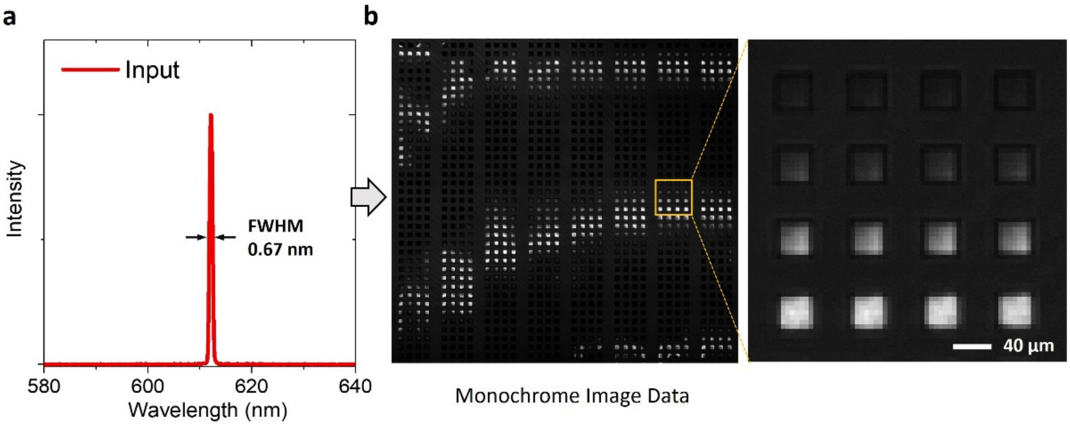

Supplementary Fig. 8 | Typical image data for spectrum reconstruction. a, The spectrum of an input light beam. **b,** Monochrome image data received by the CMOS image sensor. The scale bar in b(ii) is 40 µm.

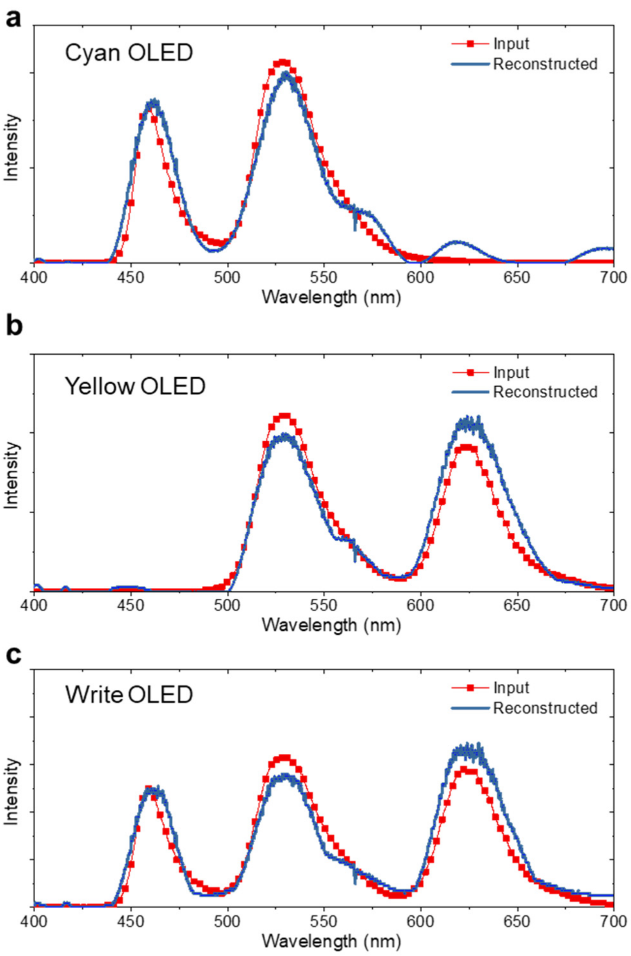

Supplementary Fig. 9 | Testing results for the measurement of the broad light spectra of three different types of OLEDs. **a**, Yellow OLED. **b**, Cyan OLED. **c**, White OLED (from the AMOLED screen of Motorola Edge 20 Pro). The corresponding cosine similarities are 0.9774, 0.9693 and 0.9630, respectively.

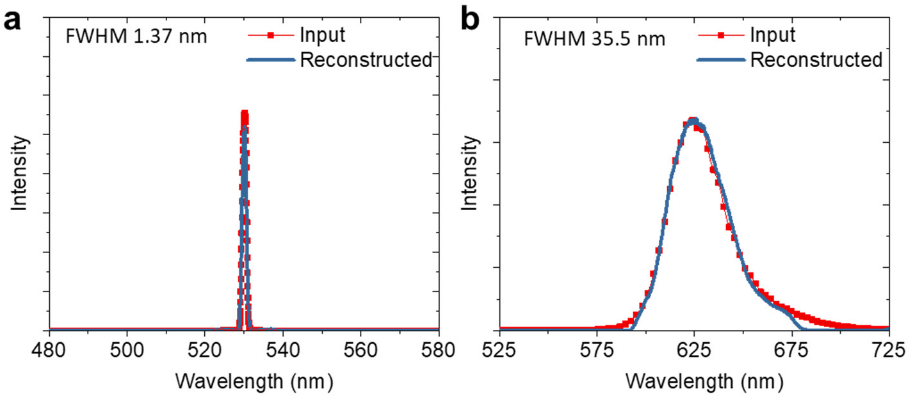

Supplementary Fig. 10 | Example of the reconstruction of narrowband (a) and broadband (b) input spectra using the same set of narrowband single-peak training spectra. The same training dataset but different hyperparameters are used to reconstruct these two input spectra. For the narrowband input spectrum (FWHM=1.37 nm), the hyperparameter settings are c_1 = 600, c_2 = 0.1, $c_{3,1}$ = 0.1, $c_{3,5}$ = 0.02, $c_{3,9}$ = 0.05, and $c_{4,1}$ = 0.06. For the broadband input spectrum (FWHM=35.5 nm), the hyperparameter settings are $c_1 = 5.8\times10^4$, c_2 = 1900, $c_{3,1} = 9.86\times10^4$, $c_{3,5} = 1.59\times10^7$, $c_{3,9}$ = 0.1, and $c_{4,1}$ = 9430. The other hyperparameters, i.e., $c_{3,p}$ (p=2, 3, 4, 6, 7, 8, or $p \geqslant 10$) and $c_{4,q}$ ($q \geqslant 2$), are all zero. The cosine similarities of the two reconstructed spectra are 0.9985 and 0.9977, respectively.

Supplementary Table 1. Comparison with recently reported computational spectrometers.

Publication information	Working mechanism	CMOS/CCD or other detectors	Operation range	Spectral resolution
Compact spectrometer based on a disordered photonic chip [1] (*Nature Photonics 2013*)	Plasmonic nanohole	Photodetector array	1500 ~ 1525 nm	0.75 nm
A colloidal quantum dot spectrometer [2] (*Nature 2015*)	Perovskite quantum dot	CCD sensor (Transmission mode)	390 ~ 690 nm	3.2 nm
Single-nanowire spectrometers [3] (*Science 2019*)	Single compositionally engineered nanowire	Nanowire detector array	500 ~ 630 nm	7 ~ 8.5 nm
Single-shot on-chip spectral sensors based on photonic crystal slabs [4] (*Nature Communications 2019*)	Photonic crystal slabs	CMOS sensor (Transmission mode)	550 ~ 750 nm	1 nm
Compact CMOS spectral sensor for the visible spectrum [5] (*Photonics Research 2019*)	Photonic crystal slabs	CMOS sensor (Transmission mode)	400 ~ 700 nm	1 nm
Broadband perovskite quantum dot spectrometer beyond human visual resolution [6] (*Light: science & applications 2020*)	Perovskite quantum dot	CCD sensor (Transmission mode)	250 ~ 1000 nm	1.6 nm
Neural network-based on-chip spectroscopy using a scalable Plasmonic Encoder [7] (*ACS Nano 2021*)	Plasmonic nanohole	CMOS sensor (Transmission mode)	480 ~ 750 nm	<3.29 nm
A pearl spectrometer [8] (*Nano Lett. 2021*)	Naturally pearl nanostructure	CCD sensor (Transmission mode)	450 ~ 700 nm	7.4 nm
Deeply learned broadband encoding stochastic hyperspectral imaging [9] (*Light: science & applications 2021*)	Multilayer film filters (using SiO_2 and TiO_2 nanolayers)	CCD sensor (Transmission mode)	400 ~ 700 nm	5.2 nm
A wavelength-scale black phosphorus spectrometer [10] (*Nature Photonics 2021*)	Tunable black-phosphorus detector	Black phosphorus detector	2000 ~ 9000 nm	420 nm
3D-printed miniature spectrometer for the visible range with a 100 × 100 μm^2 footprint [11] (*Light: Advanced Manufacturing 2021*)	3D-printed micro-optics	CMOS sensor (Transmission mode)	490 ~ 690 nm	9.2 nm
A single-dot perovskite spectrometer [12] (*Advanced Materials 2021*)	LiCl-doped perovskite film	Perovskite detector	350 ~ 750 nm	5.3 nm
Mass production-enabled computational spectrometers based on multilayer thin films [13] (*Scientific Reports 2022*)	Multilayer film filters (using SiO_2 and TiO_2 nanolayers)	CMOS sensor (Transmission mode)	500 ~ 849 nm	1 nm
Ultraspectral imaging based on metasurfaces with freeform shaped meta-atoms [14] (*Laser & Photonics Reviews 2022*)	Metasurfaces with freeform shaped meta-atoms	CMOS sensor (Transmission mode)	460 ~ 740 nm	0.5 nm
Dynamic brain spectrum acquired by a real-time ultraspectral imaging chip with reconfigurable metasurfaces [15] (*Optica 2022*)	Reconfigurable metasurfaces	CMOS sensor (Transmission mode)	450 ~ 750 nm	0.8 nm
Short-wave infrared chip-spectrometer by using laser direct-writing grayscale lithography [16] (*Advanced Optical Materials 2022*)	Directly written Fabry–Pérot cavities	Photodetector array	900 ~ 1700 nm	2 nm

Miniaturized spectrometers with a tunable van der Waals junction [17] (*science 2022*)	Tunable van der Waals junction	MoS_2/WSe_2 heterojunction detector	405 ~ 845 nm	3 nm
Deep learning-based miniaturized all-dielectric ultracompact film spectrometer [18] (*ACS Photonics 2023*)	Five-layer film stacks (using SiO_2 and TiO_2 nanolayers)	CMOS sensor (Transmission mode)	400 ~ 700 nm	5 nm (16 filters) or 3nm (64 filters)
Video-rate hyperspectral camera based on a CMOS-compatible random array of Fabry–Pérot filters [19] (*Nature photonics 2023*)	Fabry–Pérot filter array	CMOS sensor (Transmission mode)	400 ~ 700 nm	~20 nm
Imaging-based intelligent spectrometer on a plasmonic rainbow chip [20] (*Nature Communications 2023*)	Plasmonic rainbow chip	CCD sensor (Reflection mode)	400 ~ 800 nm	2 nm
Folded digital meta-lenses for on-chip Spectrometer [21] (*Nano Letter 2023*)	Folded digital meta-lenses	Photodetector	1530 ~ 1565 nm	0.14 nm
Inverse-designed linear coherent photonic networks for high-resolution spectral reconstruction [22] (*ACS Photonics 2023*)	Integrated photonic chip	Photodetector	1515 ~ 1525 nm	0.1 nm
Integrated single-resonator spectrometer beyond the free-spectral-range limit [23] (*ACS Photonics 2023*)	Single micro-ring resonator	Photodetector	1500 ~ 1600 nm	0.08 nm
Photon counting reconstructive spectrometer combining metasurfaces and superconducting nanowire single-photon detectors [24] (*Photonics Research 2023*)	Metasurface array	Nanowire single-photon detectors	1500 ~ 1600 nm	2 nm
**This work**	Plasmonic nanoparticles-in-cavity microfilter array	CMOS sensor (Transmission mode)	395 ~ 725 nm	0.65 nm (0.49 nm for 580 ~ 680 nm)

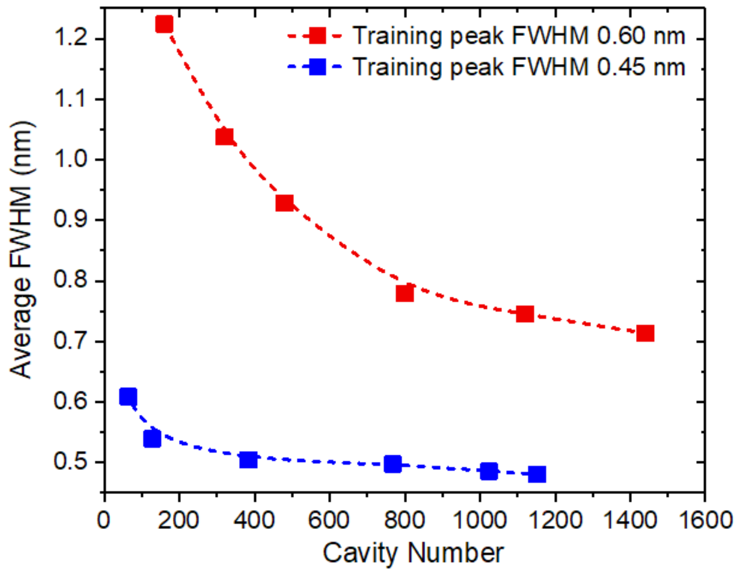

Supplementary Fig. 11 | Testing results for the effect of the microfilter number on the spectrometer resolution. Two groups of single-peak spectra with FWHMs of 0.61 nm (red) and 0.44 nm (blue) were used in the training and testing of the spectrometer.

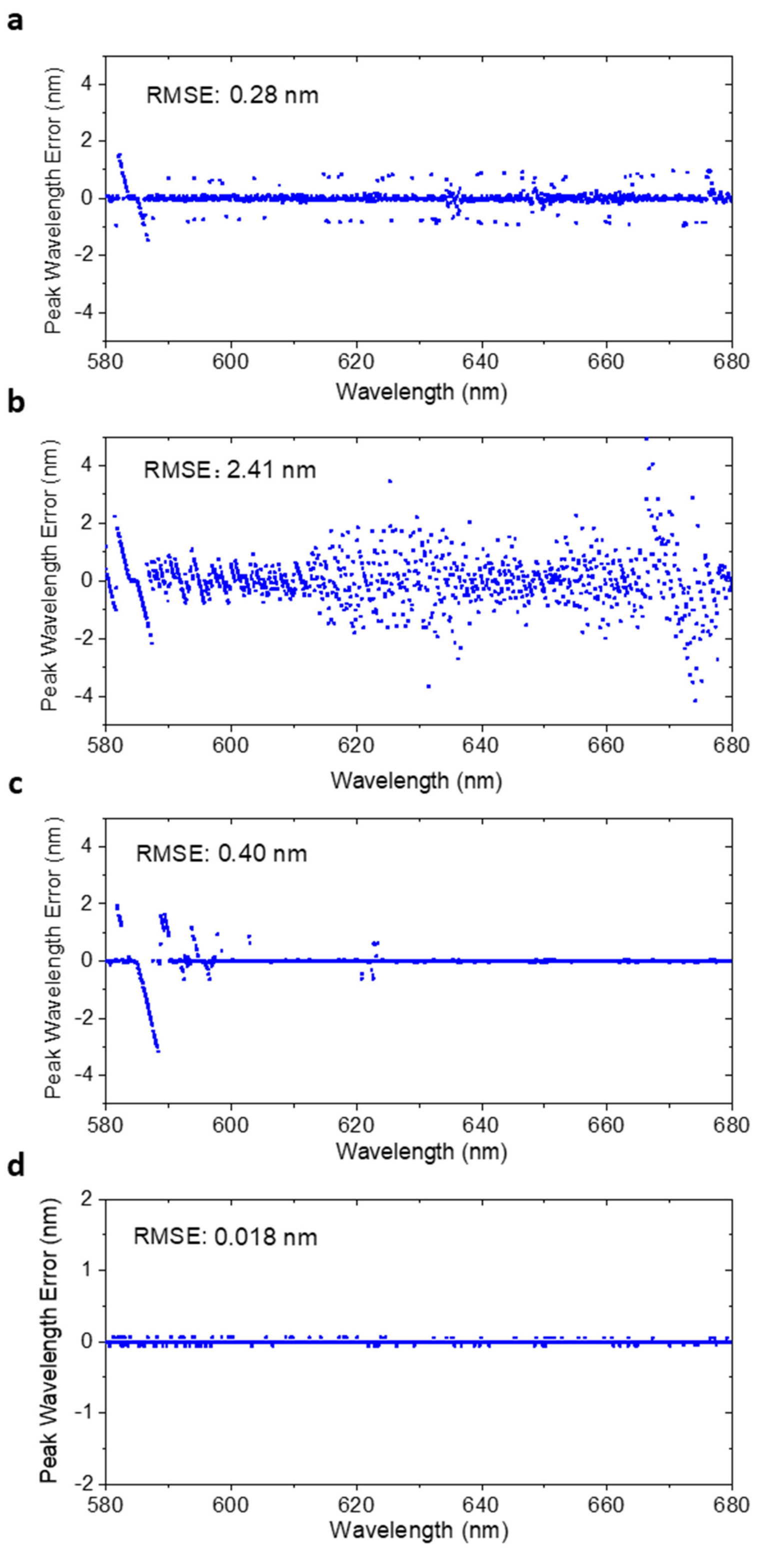

Supplementary Fig. 12 | Peak wavelength errors of the single-peak optical spectra reconstructed by using different algorithms. **a,** Ridge, **b,** TV, **c,** LASSO, **d,** Hybrid algorithm**.**

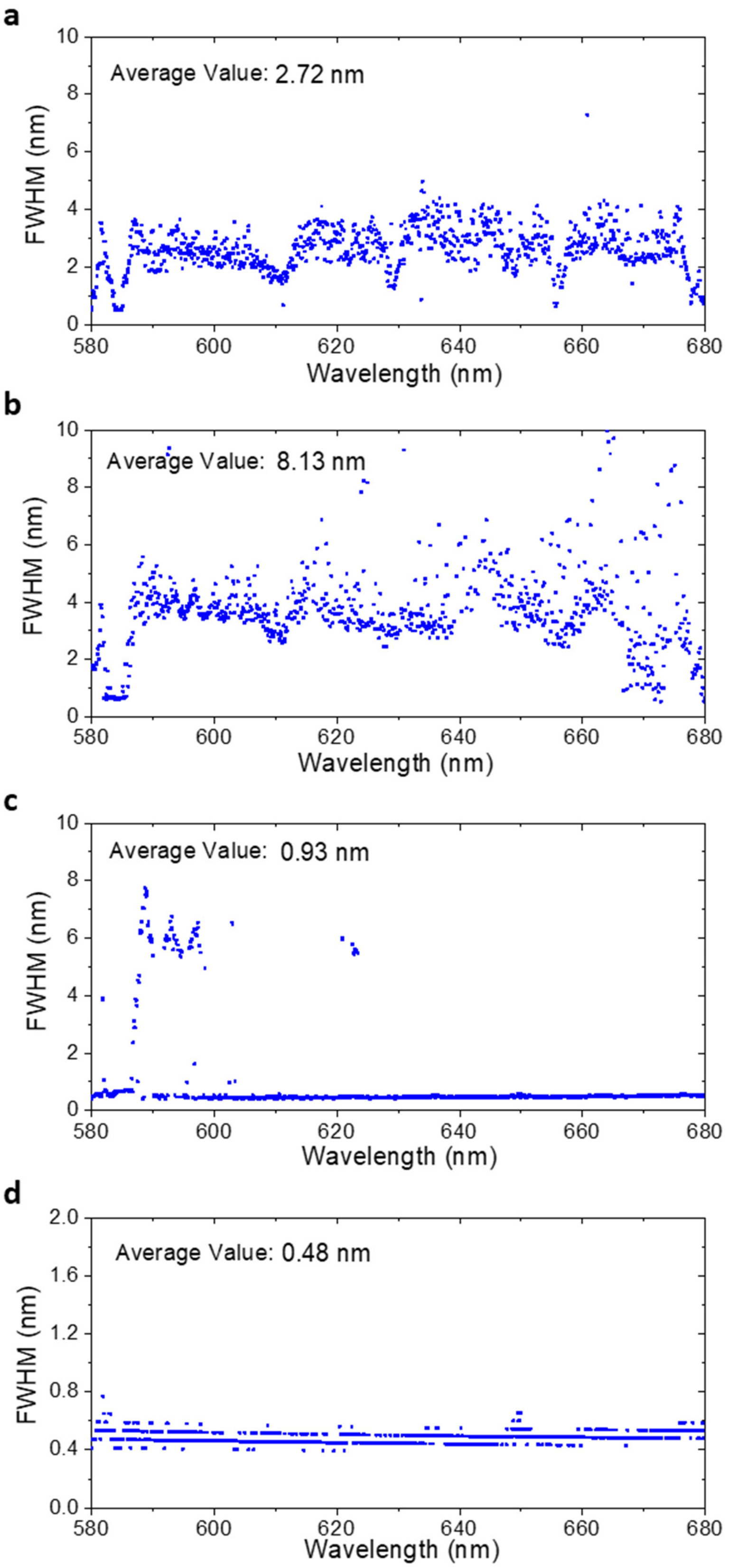

Supplementary Fig. 13 | Calculated FWHMs of single-peak optical spectra reconstructed by using different algorithms. **a,** Ridge, **b,** TV, **c,** LASSO, **d,** Hybrid algorithm.

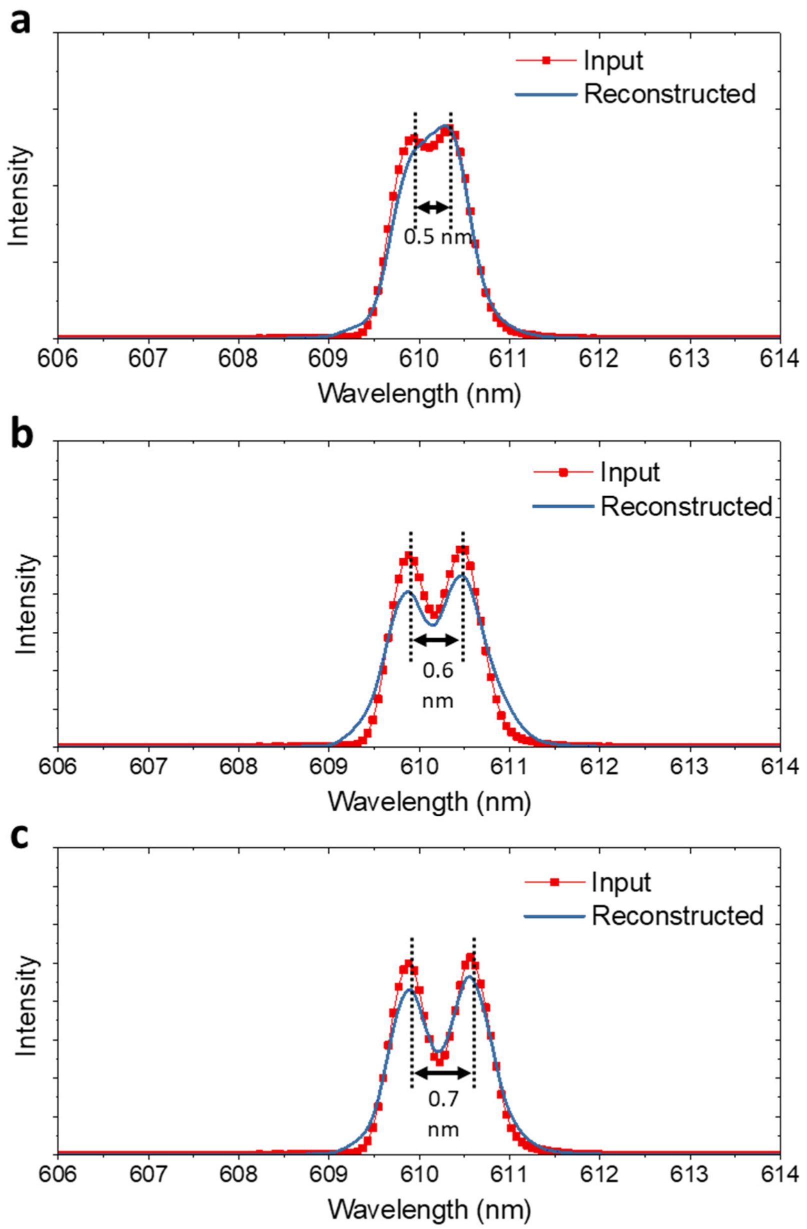

Supplementary Fig. 14 | Spectral resolution testing of the computational spectrometer by using two-peak input spectra. a, Results for two spectral peaks with a wavelength separation of 0.5 nm. **b,** Results for two spectral peaks with a wavelength separation of 0.6 nm. **c,** Results of two spectral peaks with a wavelength separation of 0.7 nm. The results were achieved by using a series of input spectra with ~0.44 nm FWHM as training data scanned with a 0.1 nm step.

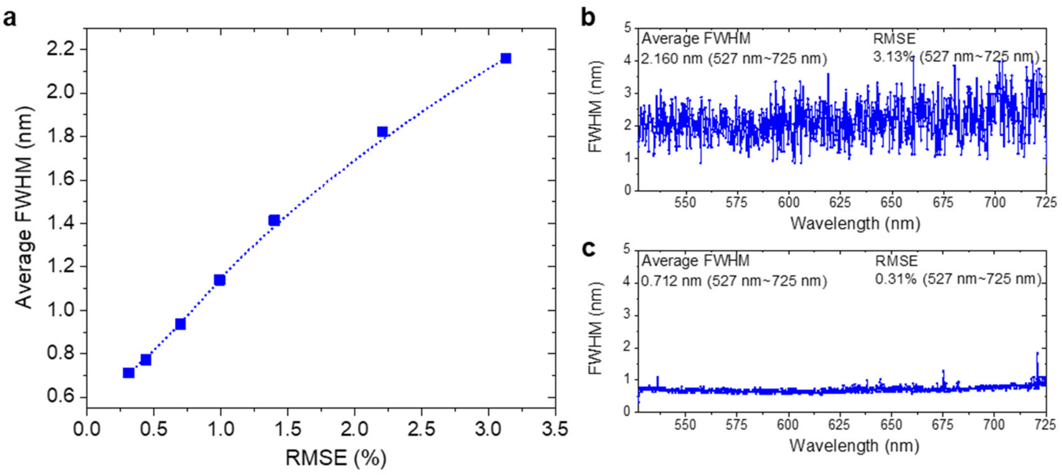

Supplementary Fig. 15 | Testing results for the effect of measurement noise on the spectrometer resolution. a, Average FWHM as a function of the RMSE. **b**, Reconstruction result with an RMSE of 3.13%. **c**, Reconstruction result with an RMSE of 0.31%. The spectrometer was trained with single-peak light spectra with a FWHM of 0.61 nm in the 525~675 nm range.

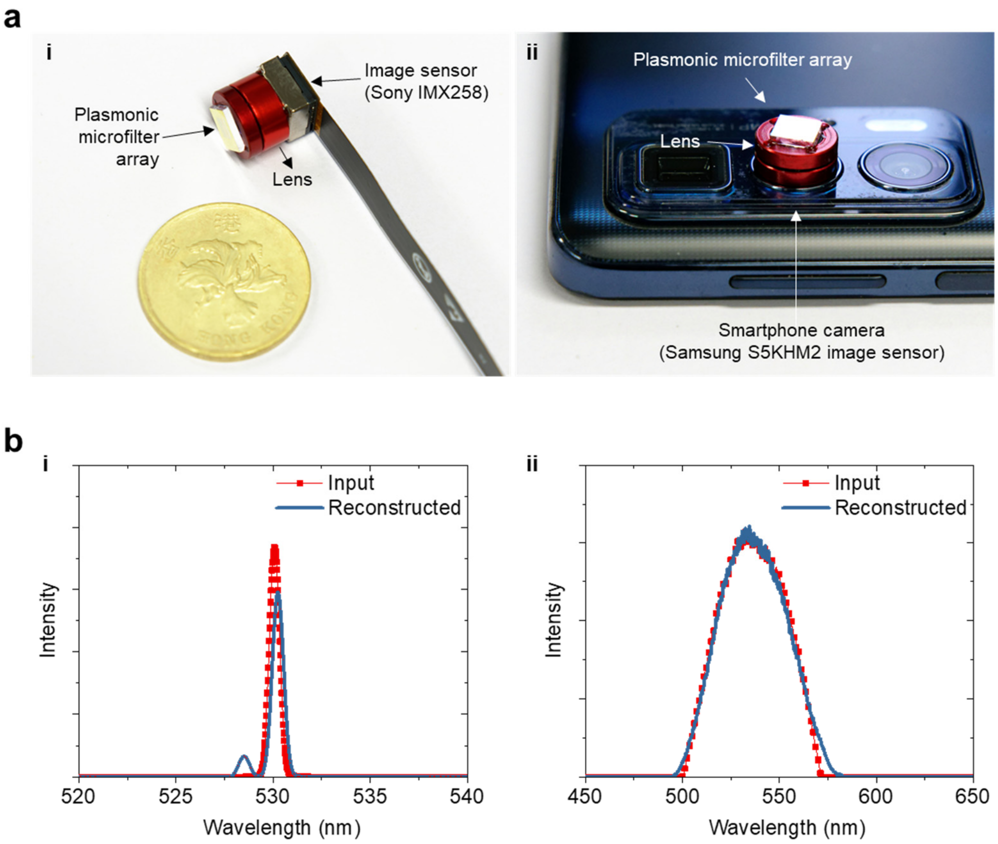

Supplementary Fig. 16 | Demonstration and testing of CMOS image sensor-based portable spectrometers with plasmonic microfilter arrays. a, Photos of the CMOS image sensor-based spectrometer (i) and the smartphone-based portable spectrometer (ii). **b**, Results of the homemade portable spectrometer shown in **a**(i) for the measurement of a single-narrow-peak spectrum (i) and broadband light input (ii).

Supplementary References:

1. Redding B., Liew S.F., Sarma R., Cao H. Compact spectrometer based on a disordered photonic chip. *Nat. Photonics* 2013, **7**(9)**:** 746-751.
2. Bao J., Bawendi M.G.. A colloidal quantum dot spectrometer. *Nature* 2015, **523**(7558)**:** 67-70.
3. Yang Z., Albrow-Owen T., Cui H., Alexander-Webber J., Gu F., Wang X*., et al.* Single-nanowire spectrometers. *Science* 2019, **365**(6457)**:** 1017-1020.
4. Wang Z., Yi S., Chen A., Zhou M., Luk T.S., James A*., et al.* Single-shot on-chip spectral sensors based on photonic crystal slabs. *Nat. Commun.* 2019, **10**(1)**:** 1020.
5. Zhu Y, Lei X, Wang KX, Yu Z. Compact CMOS spectral sensor for the visible spectrum. *Photonics Research* 2019, **7**(9): 961-966.
6. Zhu X., Bian L., Fu H., Wang L., Zou B., Dai Q*., et al.* Broadband perovskite quantum dot spectrometer beyond human visual resolution. *Light Sci. Appl.* 2020, **9**(1)**:** 73.
7. Brown C., Goncharov A., Ballard Z.S., Fordham M., Clemens A., Qiu Y*., et al.* Neural network-based on-chip spectroscopy using a scalable plasmonic encoder. *ACS nano* 2021, **15**(4)**:** 6305-6315.
8. Kwak Y., Park S.M., Ku Z., Urbas A., Kim Y.L. A pearl spectrometer. *Nano Lett.* 2020, **21**(2)**:** 921-930.
9. Zhang W., Song H., He X., Huang L., Zhang X., Zheng J*., et al.* Deeply learned broadband encoding stochastic hyperspectral imaging. *Light Sci. Appl.* 2021, **10**(1)**:** 108.
10. Yuan S., Naveh D., Watanabe K., Taniguchi T., Xia F.. A wavelength-scale black phosphorus spectrometer. *Nat. Photonics.* 2021, **15**(8)**:** 601-607.
11. Toulouse A., Drozella J., Thiele S., Giessen H., Herkommer A. 3D-printed miniature spectrometer for the visible range with a 100× 100 μm 2 footprint. *Light Adv. Manuf.* 2021, **2**(1): 20-30.
12. Guo L., Sun H., Wang M., Wang M., Min L., Cao F*., et al.* A Single - Dot Perovskite Spectrometer. *Adv. Mater.* 2022, **34**(33)**:** 2200221
13. Kim C., Ni P., Lee K.R,. Lee H.N.. Mass production-enabled computational spectrometers based on multilayer thin films. *Sci. Rep.* 2022, **12**(1): 4053.
14. Yang J., Cui K., Cai X., Xiong J., Zhu H., Rao S*., et al.* Ultraspectral imaging based on metasurfaces with freeform shaped meta-atoms. *Laser Photonics Rev.* 2022, **16**(7)**:** 2100663.
15. Xiong J., Cai X., Cui K., Huang Y., Yang J., Zhu H*., et al.* Dynamic brain spectrum acquired by a real-time ultraspectral imaging chip with reconfigurable metasurfaces. *Optica* 2022, **9**(5)**:** 461-468.
16. Xuan Z, Wang Z, Liu Q, Huang S, Yang B, Yang L, et al. Short - Wave Infrared Chip - Spectrometer by Using Laser Direct - Writing Grayscale Lithography. Adv Opt Mater 2022, 10(19): 2200284.
17. Yoon H.H., Fernandez H.A., Nigmatulin F., Cai W., Yang Z., Cui H*., et al.* Miniaturized spectrometers with a tunable van der Waals junction. *Science* 2022, **378**(6617)**:** 296-299.
18. Wen J, Hao L, Gao C, Wang H, Mo K, Yuan W, et al. Deep Learning-Based Miniaturized All-Dielectric Ultracompact Film Spectrometer. *ACS Photonics* 2022, **10**(1): 225-233.
19. Yako M, Yamaoka Y, Kiyohara T, Hosokawa C, Noda A, Tack K, et al. Video-rate hyperspectral

camera based on a CMOS-compatible random array of Fabry–Pérot filters. *Nat. Photonics* 2023, **17**(3): 218-223.

20. Tua, D., Liu, R., Yang, W., Zhou, L., Song, H., Ying, L., Gan, Q.. Imaging-based intelligent spectrometer on a plasmonic rainbow chip. *Nat. Commun.* 2023, **14**: 1902.
21. Zhang Z, Liu Y, Wang Z, Zhang Y, Guo X, Xiao S, et al. Folded Digital Meta-Lenses for on-Chip Spectrometer. *Nano Letters* 2023, **23**(8): 3459-3466.
22. Li Y, Zhang Z, Wang Y, Yu Y, Zhou X, Tsang HK, et al. Inverse-Designed Linear Coherent Photonic Networks for High-Resolution Spectral Reconstruction. *ACS Photonics* 2023, **10**(4): 1012-1018.
23. Xu H, Qin Y, Hu G, Tsang HK. Integrated single-resonator spectrometer beyond the free-spectral-range limit. *ACS Photonics* 2023, **10**(3): 654-666.
24. Zheng, J., Xiao, Y., Hu, M., Zhao, Y., Li, H., You, L*., et al.* Photon counting reconstructive spectrometer combining metasurfaces and superconducting nanowire single-photon detectors. *Photonics Research* 2023, **11**(2): 234-244.
